# Large-scale analysis of putative Euphorbiaceae R2R3-MYB transcription factors identifies a MYB involved in seed oil biosynthesis

**DOI:** 10.1186/s12870-023-04163-5

**Published:** 2023-03-17

**Authors:** Yunpeng Cao, Tingting Fan, Lihu Wang, Lin Zhang, Yanli Li

**Affiliations:** 1 CAS Key Laboratory of Plant Germplasm Enhancement and Specialty Agriculture, Wuhan Botanical Garden, Chinese Academy of Sciences, 430074 Wuhan, China; 2 School of Health and Nursing, Wuchang University of Technology, Wuhan, China; 3 Forestry College, Central South University of Forestry and Technology, 410004 Changsha, Hunan China; 4 College of Landscape and Ecological Engineering, Hebei University of Engineering, 056009 Handan, China; 5 School of Basic Medical Sciences, Hubei University of Chinese Medicine, 430065 Wuhan, China

**Keywords:** Euphorbiaceae, Evolutionary rate, MYB, Gene feature, Paralog, Ortholog

## Abstract

**Background:**

MYB transcription factors are widely distributed in the plant kingdom and play key roles in regulatory networks governing plant metabolism and biochemical and physiological processes.

**Results:**

Here, we first determined the R2R3-MYB genes in five Euphorbiaceae genomes. The three Trp (W) residues from the first MYB domain (R2) were absolutely conserved, whereas the first W residue from the second MYB domain (R3) was preferentially mutated. The R2R3-MYBs were clustered into 48 functional subfamilies, of which 34 had both R2R3-MYBs of Euphorbiaceae species and AtMYBs, and four contained only Euphorbiaceae R2R3-MYBs. The whole-genome duplication (WGD) and/or segmental duplication (SD) played key roles in the expansion of the R2R3-MYB family. Unlike paralogous R2R3-MYB family members, orthologous R2R3-MYB members contained a higher selective pressure and were subject to a constrained evolutionary rate. *VfMYB36* was specifically expressed in fruit, and its trend was consistent with the change in oil content, indicating that it might be involved in oil biosynthesis. Overexpression experiments showed that *VfMYB36* could significantly provide linolenic acid (C18:3) content, which eventually led to a significant increase in oil content.

**Conclusion:**

Our study first provides insight into understanding the evolution and expression of R2R3-MYBs in Euphorbiaceae species, and also provides a target for the production of biomass diesel and a convenient way for breeding germplasm resources with high linolenic acid content in the future.

**Supplementary Information:**

The online version contains supplementary material available at 10.1186/s12870-023-04163-5.

## Background

Transcription factors generally consist of at least four distinct domains, i.e. a nuclear localization signal, a DNA-binding domain, an oligomerization site, and a protein-protein binding domain [1, 2]. Many important biological processes are controlled by transcription factors that regulate gene expression [3–6]. MYB is considered to be the largest family of transcription factors in plants, which is involved in the regulation of many biological processes, such as development, differentiation, metabolism, and defense [7–13]. The common feature of MYB family proteins is that they all contain a highly conserved MYB DNA-binding domain [9, 11, 14]. This domain consists of three α-helixes, of which the second and third helices form a helix-turn-helix (HTH) structure, and further studies have shown that the third helix directly interacts with the main grooves of target DNA [9, 11, 14, 15]. In addition, some studies have found that there were highly divergent activation regions at the C-terminus of the *MYB* genes, which allow for extensive regulation in different biological processes [16, 17]. The *MYB* gene family can be divided into four subgroups according to the number of adjacent MYB repeats, namely 1R-MYB (MYB-related proteins), 2R-MYB (R2R3-MYB proteins), 3R-MYB (R1R2R3-MYB proteins) and 4R-MYB (4R-like MYB proteins), which have one, two, three and four MYB DNA-binding domain repeats, respectively [14, 18]. All four types of *MYBs* have been identified, of which 1R-MYB and R2R3-MYB are the most frequent in the plant kingdom [19]. Phylogenetic studies have shown that R2R3-MYBs likely evolved from R1R2R3-MYBs by losing the R1 repeat, or from R1-MYBs by duplication R1 repeat [8, 14, 19, 20].

The first identified *MYB*, *v-MYB*, was isolated from the avian myeloblastosis virus (AMV) [21]. *C1* (*NP_001106010.1*) in *Zea mays*, the first MYB identified in plants, regulates the biosynthesis of anthocyanin [22]. Based on the analysis of molecular and genetic experiments, an increasing number of R2R3-MYBs have been characterized and studied in many plants, such as pear (*Pyrus bretschneideri*), apple (*Malus domesti*c), grapevine (*Vitis vinifera*), petunia (*Petunia hybrida*), rice (*Oryza sativa*), poplar (*Populus tremuloide*s), maize (*Zea may*) and *Arabidopsis thaliana* [7, 18, 20, 23]. For example, *Lycopersicon esculentum* and *A. thaliana MYB15* plays a negative regulatory role in the CBF pathway in response to cold stress [24, 25]. *PbMYB25* and *PbMYB52* from *P. bretschneideri* are considered to be regulators of lignin biosynthesis during fruit development [7]. The *O. sativa OsMYB2* gene has been shown to act as a potentially important regulator in tolerance to dehydration and salt and cold stresses [26]. *PtrMYB3* and *PtrMYB20* from *P. tremuloide*s control the lignin biosynthesis by upregulating the monolignol pathway [27]. In 2015, Li et al. reported that *Populus tomentosa PtoMYB92* as a transcriptional activator regulates the biosynthesis of lignin during secondary cell wall formation [28].

Members of R2R3-MYB family are involved in multiple plant-specific processes, raising the hypothesis that their expansion may be the cause of plant evolutionary diversity. The R2R3-MYBs have been analyzed and identified from several sequenced plants, such as pear, apple, wheat, rice, poplar, *Beta vulgaris*, rice and *A. thaliana*. Most studies were focused on MYB family identification and gene structure, phylogenetic, conserved domain, and chromosomal localization analyses. Additionally, the functions of some R2R3-MYBs have been analyzed by molecular biological experiments in model plants [4, 14]. However, the predicted functions for each subclade of the R2R3-MYB family and potential evolutionary mechanisms in the R2R3-MYB family are still uncertain.

Euphorbiaceae species including tung tree (*Vernicia fordii*), rubber tree (*Hevea brasiliensis*), physic nut (*Jatropha curcas*), cassava (*Manihot esculenta*), and castor oil plant (*Ricinus communis*) are economically important plants. These Euphorbiaceae genomes have been sequenced and published. Although the in-depth study of the R2R3-MYB family has been carried out in some model plants including poplar and *A. thalian*a, systematic analysis of the R2R3-MYB family was lacking in Euphorbiaceae. Here, we carried out a genome-wide analysis of the R2R3-MYB family to identify R2R3-MYB members and investigated family evolutionary patterns, selection pressures, and gene duplication types in five Euphorbiaceae genomes. Additionally, compared with the other four species of the family Euphorbiaceae, *V. fordii* produces tung oil containing a high proportion (~ 80%) of eleostearic acid and has more than 1,000 years of cultivation history in China as an ornamental plant or for the production of tung oil [29–31]. Therefore, we selected the R2R3-MYB gene from *V. fordii* for further expression analysis, aiming at mining candidate genes related to oil synthesis. Our study might help us to understand the evolution and expansion of the R2R3-MYBs in Euphorbiaceae genomes based on the comparative analysis, and also provide valuable candidate *VfMYB36* for improving oil production by marker-assisted breeding in *V. fordii*.

## Results and discussion

### R2R3-MYBs in Euphorbiaceae

**Fig. 1:**
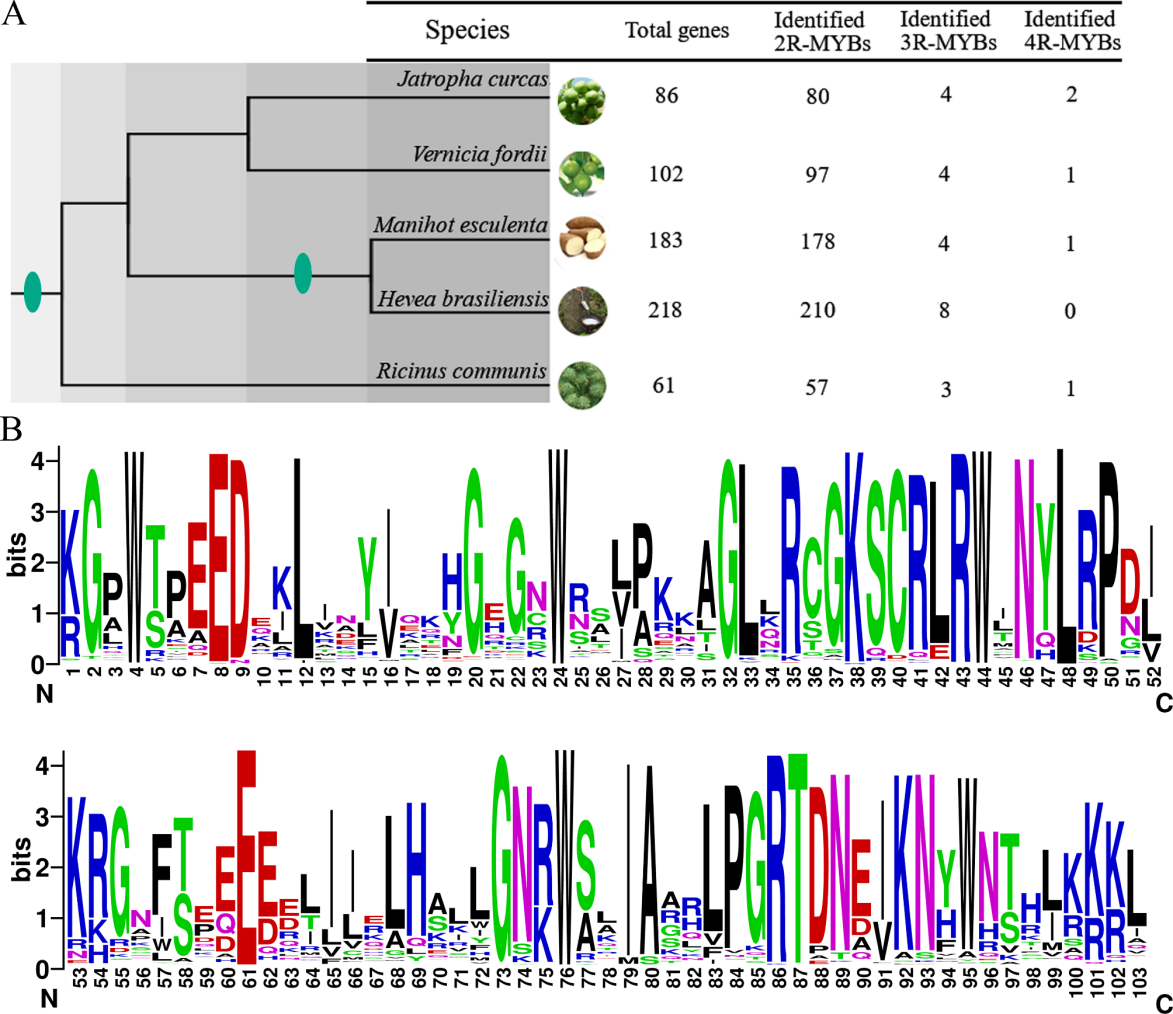
The species tree (**A**) and domain composition (**B**) of five Euphorbiaceae genomes for identified *MYB* genes. **A**, the species tree was obtained from NCBI CommonTree (https://www.ncbi.nlm.nih.gov/Taxonomy/CommonTree/wwwcmt.cgi) and Timetree (http://www.timetree.org/). The whole genome duplication (WGD) was indicated by the green oval. The numbers on the right indicated the number of corresponding genes in each genome. **B**, the logos of R2 and R3 domains were generated from the multiple alignment analysis of all R2R3-MYBs

It is well known that R2R3-MYBs can control many plant-specific processes, such as abiotic stress tolerance, plant development, disease resistance, and hormone signal transduction [8–10, 14, 17, 23, 32]. As an important family of transcription factors, R2R3-MYB members are widely present in plants, such as 129 R2R3-MYB genes in pear [7], 222 in apple [33], 192 in Poplar [34], 102 in rice [35], 55 in cucumber [36], 244 in soybean [37], 118 in grape [38], 157 in maize [23] and 124 in *A. thaliana* [18]. To obtain the R2R3-MYBs, we searched the entire genomes of *R. communis*, *J. curcas*, *M. esculenta*, *H. brasiliensis*, and *V. fordii* that encode proteins containing MYB domain (PF000249) by HMMER v3.2.1. At the same time, the *A. thaliana* R2R3-MYBs were also used as queries to perform BlastP against Euphorbiaceae genomes. The SMART, Pfam, and InterPro databases were used to examine the presence or completeness of MYB domain (PF000249). Finally, we identified 650 MYB genes that encode proteins containing at least two MYB domains (Fig. 1A and Table S1). Among them, 80 typical R2R3-MYBs, four 3R-MYBs, and two 4R-MYBs were scanned in *J. curcas*, 97 typical R2R3-MYBs, four 3R-MYBs, and one 4R-MYB were identified in *V. fordii*, 178 typical R2R3-MYBs, four 3R-MYBs and one 4R-MYB were identified in *M. esculenta*, 210 typical R2R3-MYBs, and eight 3R-MYBs were found in *H. brasiliensis*, and 57 typical R2R3-MYBs, three 3R-MYBs and one 4R-MYB were found in *R. communis*. We named *MYBs* from *J. curcas*, *R. communis*, *M. esculenta*, *H. brasiliensis* and *V. fordii* based on the order of these gene locations among the chromosomes. Here, only the primary transcript was retained when the alternative splicing events were detected for the *MYB* genes. A comparison of the number of R2R3-MYBs among these Euphorbiaceae species suggested that both *M. esculenta* and *H. brasiliensis* had the greatest number of R2R3-MYBs, while *R. communis* possessed the fewest R2R3-MYBs (Fig. 1A).

The frequency of amino acid sequences in the R2 and R3 repeats was investigated by examining the characteristics of the conserved domain of R2R3-MYB (Fig. 1B). The results showed that the MYB domains are highly conserved and they have few insertions or deletions other than those containing basic residues, which were consistent with those of cucumber, pear, soybean, sugar beet, poplar, grape, maize, rice, *Cyclocarya paliurus*, and *A. thaliana* [7, 15, 23, 39]. The R2R3-MYB domain that contained common sequences was [-W-(X19)-W-(X19)-W-. … .-F/I-(X18)-W-(X18)-W-], which formed the primary structure of R2 and R3 repeats (Fig. 1B). Subsequently, we found that the first tryptophan (W) residue of the R3 repeat was usually replaced by hydrophobic amino acids, which was a common phenomenon in plant R2R3-MYBs [14, 18, 23]. It has been observed that the substitution of hydrophobic amino acids for tryptophan residues in animal MYB domains does not result in a significant loss of DNA-binding activity [40]. This observation suggests that the hydrophobic residues can substitute for tryptophan and maintain the function of the MYB domain, at least in terms of DNA-binding. Therefore, this change in Euphorbiaceae species might also have little or no effect on the DNA-binding activity of MYB domains. Besides the highly conserved W residues, G^32^, R^43,^ and L^48^ in the R2 repeat, E^61^, G^73,^ and T^87^ in the R3 repeat were also completely conserved in these MYBs from five Euphorbiaceae species. In summary, as reported in plants such as pear, apple, rice and *A. thaliana* [7, 18, 33, 35], the conserved amino acid residues mainly occurred in the second and third conserved tryptophan residues of each MYB domain (i.e., R2 or R3).

### Synteny tests suggest the origin and expansion of the R2R3-MYBs in Euphorbiaceae

In plants, the common view of most studies is that R2R3-MYBs evolved from the R1R2R3-MYBs by losing a R1 domain and subsequent expansion of this gene family [41, 42]. Despite recent diversification events, several gene duplications, including tandem duplication (TD), whole-genome duplication (WGD) or segmental duplication (SD), and rearrangements, can drive the evolution of gene family members [43, 44]. To further understand which duplications were involved in the *MYBs*, we analyzed the synteny regions for this gene family among Euphorbiaceae genomes. Subsequently, all *MYB* members were assigned to five distinct duplication events: TD, WGD or SD, dispersed duplication (DD), proximal duplication (PD), and singleton. Results presented that DD and WGD or SD events were the main modes in these tested Euphorbiaceae genomes, and the TD event also contributed to the expansion of the *MYB* family. However, we noted that the *RcMYBs* and *VfMYBs* were not involved in the PD event. These results showed that the *MYB* members were involved in different duplication models, and DD and WGD or SD had a relatively high frequency in five tested Euphorbiaceae genomes (Figure S1). Previous investigations have demonstrated that the WGD or SD event is the primary driver of *MYBs* expansion in soybean and pear [37, 45]. Combining previous reports with our study, we argue that WGD or SD events should be the most important contributors to the expansion of *MYB* members in plants.

**Fig. 2:**
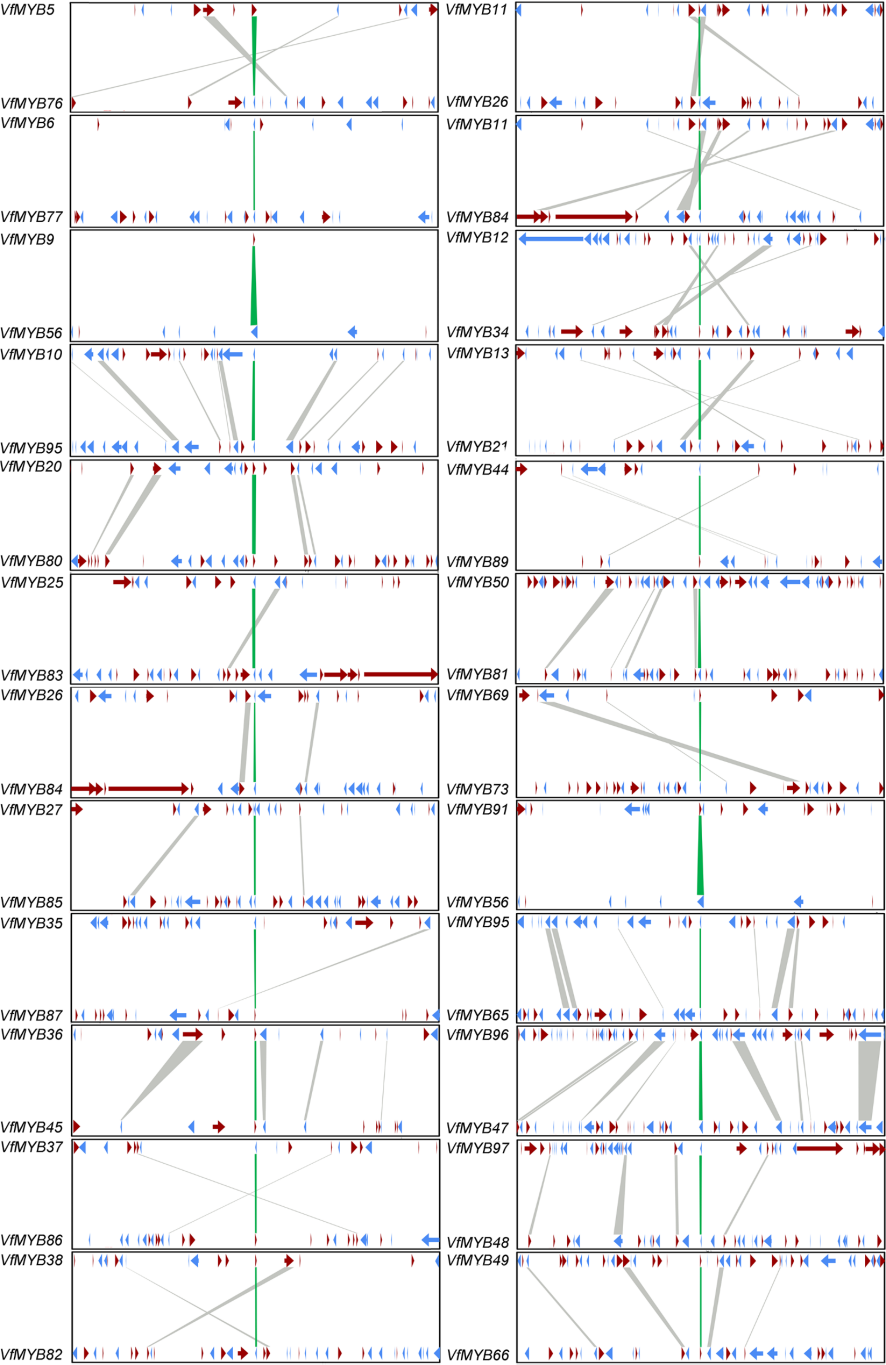
Synteny blocks of *VfMYBs* in *V. fordii*. The position of the *VfMYB* was highlighted in green and the relative positions of the other flanking genes were determined by the position of the *VfMYB*. The lines indicated that the genes have synteny relationships, and the arrows represented the transcriptional orientations of these genes

To infer the evolutionary mechanism of the *MYB* family in five Euphorbiaceae genomes, a process similar to PGDD [46] for scanning the synteny blocks was used in this study. Totally, 25, 4, 123, 41 and 13 segmentally duplicated *MYB* pairs were identified in the *V. fordii*, *R. communis*, *M. esculenta*, *H. brasiliensis* and *J. curcas* genomes (Table S2). The homologs in the 100 kb flanking on each side of the anchored *MYBs* were also searched (Table S3). For example, in duplicated *VfMYB50-VfMYB81* pair, the number of genes in a 200 Kb window is up to 92, while the homologs in the 100 kb flanking each side of *VfMYB47-VfMYB96* reached 9 pairs in *V. fordii* genome (Fig. 2 and Table S2). The conserved synteny in R2R3-MYBs was also observed from other four Euphorbiaceae genomes, and these results suggested that the large-scale duplications played important roles in the expansion of the R2R3-MYB family in these tested genomes.

*R. communis*, *H. brasiliensis, J. curcas*, *M. esculenta* and *V. fordii* shared an ancient WGD event (i.e. γ-triplication; Ks ∼1.08–1.48), while both of *M. esculent* and *H. brasiliensis* also experienced an additional recent WGD event (i.e. ∼15.3 Myr ago; Ks ∼0.2–0.37) [47, 48]. Thus, the synonymous substitutions per site (Ks) was further calculated to determine the evolutionary dates of WGDs or SDs associated with *MYB* genes from these five Euphorbiaceae genomes (Table S4), suggesting that *MYB* duplicated genes may be derived from the ancient WGD event and/or recent WGD event. At the same time, we also noted that the recent WGD event was attributed to the expansion of R2R3-MYB family members in both the *H. brasiliensis* and *M. esculenta* than the ancient WGD event. These results explained that there are far more members of the R2R3-MYB family in *M. esculenta* and *H. brasiliensis* than in the other three Euphorbiaceae, and also indicated that R2R3-MYB is an ancient family whose members expanded with the recent WGD.

### Phylogenetic relationships among five Euphorbiaceae species

**Fig. 3:**
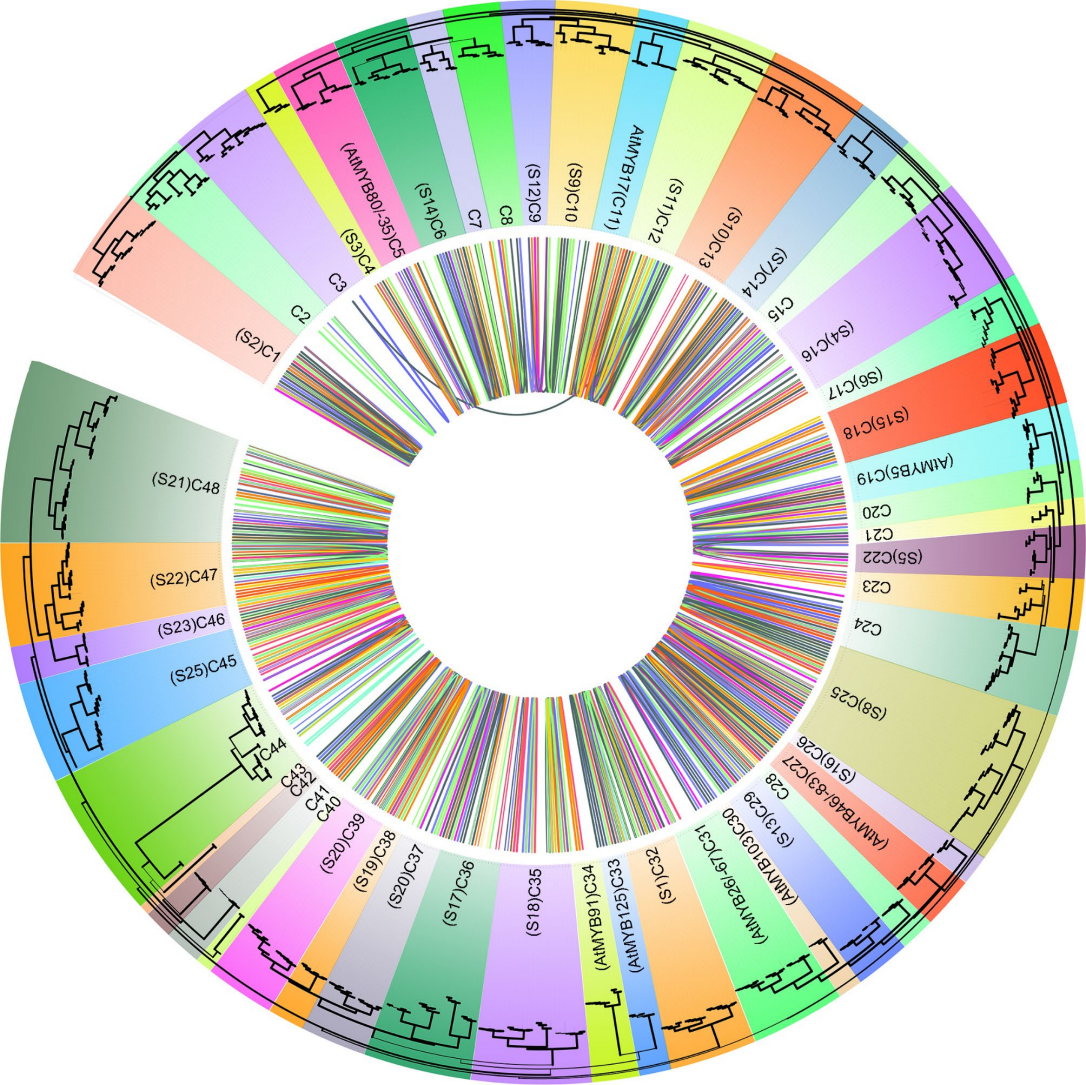
Synteny and phylogenetic tree analysis among these five Euphorbiaceae species. Synteny relationships were indicated by different links among these five Euphorbiaceae species. The ML tree was constructed using IQ-tree with the best substitution model

To test the phylogeny within the R2R3-MYB family, all identified R2R3-MYBs from the five Euphorbiaceae genomes were aligned with 126 *AtMYB* genes. *AtMYB* family has been determined in *A. thaliana* [18], and the family members were involved in various plant biochemical and physiological processes [8, 10, 14]. The previous results for *AtMYB* genes were also considered in our subfamily classification of the R2R3-MYBs from the five Euphorbiaceae to confirm the result of our phylogenetic reconstruction. The phylogenetic allowed us to classify the R2R3-MYBs from five Euphorbiaceae into 48 clades (C1–C48) being supported by collinearity analysis (Fig. 3 and Figure S2), indicating that these genes in each clade may possess similar functions and evolve from the same duplicated event. According to the conservation of the amino acid motifs and DNA-binding domain, Stracke et al. (2001) have divided the *A. thaliana* R2R3-MYBs into 25 subgroups [18]. Compared with our classification of the R2R3-MYBs from Euphorbiaceae genomes, the subgroup (S) classification of *A. thaliana* was also applied to these species. Here, we clustered 48 clades, including 14 species-specific R2R3-MYB clades among the five Euphorbiaceae genomes for which there were no representatives in *A. thaliana*, indicating these genes might play special roles that were either gained in these tested five species or lost in *A. thaliana* after divergence from the last common ancestor (Fig. 3). We also noted that the remaining *A. thaliana* R2R3-MYBs have not emerged in any subgroup in *A. thaliana* (Fig. 3). These *A. thaliana* R2R3-MYB genes were divided into eight subfamilies with Euphorbiaceae R2R3-MYBs. For instance, the subfamily C23 contained *AtMYB26*, *AtMYB67* and *10 RcMYBs, 13 VfMYBs, 2 HbMYBs*, indicating that the functional roles of these gene family members further differentiated, and the R2R3-MYB family further expanded in plants.

The phylogenetic tree suggested that R2R3-MYB family members from the five Euphorbiaceae species were unevenly distributed within given subfamilies. For example, the subfamily C23 included two, three, and four R2R3-MYB genes from *R. communis*, *J. curcas*, and *V. fordii*, while it had eight and nine R2R3-MYB genes from *M. esculenta* and *H. brasiliensis*, respectively (Fig. 3). These phenomena were also consistent with our knowledge that the *J. curcas*, *R. communis*, and *V. fordii* have not undergone the recent WGD event shared by *H. brasiliensis* and *M. esculenta* during evolution. Most of these given subfamilies had R2R3-MYB genes from the five Euphorbiaceae species, indicating that these MYBs within the given subfamilies might have already existed in their common ancestral species. We noted that several subfamilies were scanned only in several particular genomes. For example, two (C3 and C7) subfamilies were present in *H. brasiliensis* and *M. esculenta*, but not in *J. curcas* and *R. communis*. These results indicated that these genes might play specialized roles that were either acquired in *M. esculenta* and *H. brasiliensis* or lost in *R. communis* and *J. curcas*. Remarkably, four *VfMYB* genes showed ambiguous placement between different phylogenetic trees or did not divide into any of the subfamilies, indicating that these R2R3-*MYB* genes might have specialized functions that were expanded or acquired in these species during evolution.

### Evolutionary rates and gene features in homologs

**Fig. 4:**
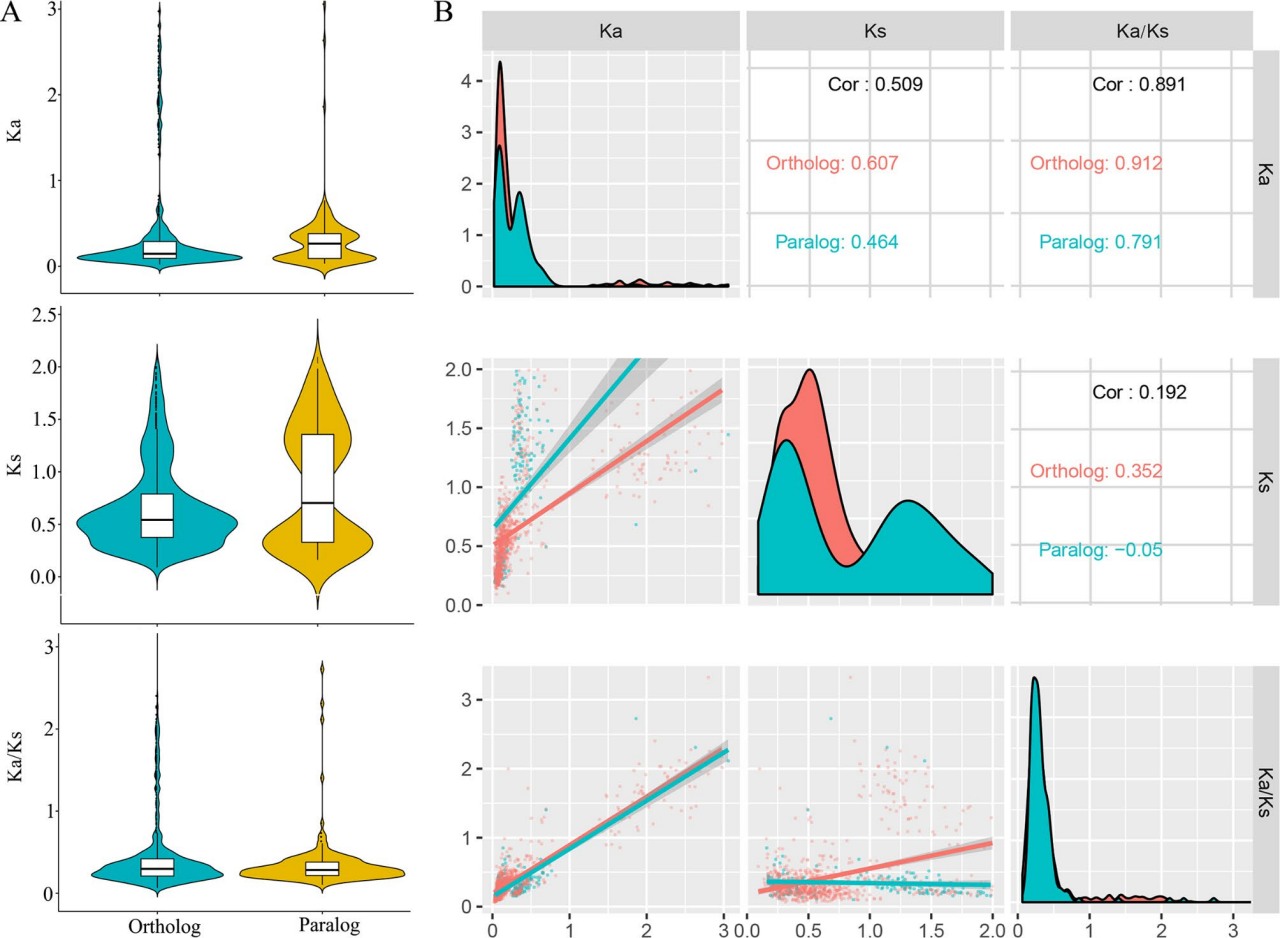
Comparison of substitution rates between orthologous and paralogous *MYB* genes among these five Euphorbiaceae species. **A**, Comparative analysis of Ka, Ks and Ka/Ks of paralogous and orthologous *MYB* genes. **B**, Correlation analysis between Ka, Ks and Ka/Ks of paralogous and orthologous *MYB* genes

In our study, we excluded these homologous genes, if the Ka value was nearly 0, and the Ks was more than 2 or less than 0.01, because a high Ks value suggested potential sequence saturation, and low sequence divergence could lead to unknown results [49]. Subsequently, we found that the average Ka, Ks, and Ka/Ks values of the paralogs were 0.29, 0.35, and 0.86, respectively. The average Ka, Ks, and Ka/Ks values of the orthologs were 0.33, 0.43, and, 0.65, respectively (Fig. 4A and Table S4). A comparison of the orthologs and paralogs suggested that the average Ka and Ka/Ks values of the paralogs were lower than that of the orthologs, but the Ks for paralogs were higher than that of the orthologs. Our data suggested that the orthologs instead of paralogs had a higher selective pressure and were subject to a constrained evolutionary rate. We also performed correlation analyses to explore the relationship between Ks, Ka, and Ka/Ks values of both orthologs and paralogs (Fig. 4B). These results revealed that the Ks values were negatively correlated with the Ka/Ks in paralogs (r= -0.05, p < 0.5), but this value was positively correlated with the Ka/Ks in orthologs (r = 0.352, p < 0.01). The positive correlation between Ka and Ks was detected in orthologs and paralogs (ortholog: r = 0.607, P < 0.01; paralog: r = 0.464, P < 0.01). At the same time, a positive correlation between Ka and Ka/Ks was found in orthologs and paralogs (ortholog: r = 0.691, P < 0.01; paralog: r = 0.912, P < 0.01). Our study suggested that the Ka/Ks values were mainly affected by Ka, but also affected to some extent by Ks in paralogs and orthologs.

Previous studies have confirmed that there is a correlation between gene features and evolutionary rate, but the correlation is different in various organisms. Here, we found that the Ks, Ka, and Ks/Ks were negatively correlated with GC1, GC2, GC3, and Fop in orthologs and paralogs, respectively (Table S5). In orthologs, the Ka, Ks, and Ka/Ks were significantly negatively correlated with GC3 and GC1, respectively, except for Ks with GC3. In paralogs, the Ka, Ks, and Ks/Ks were also negatively correlated with GC1 and GC3, but not significantly with GC3. Our data suggested that GC1 influenced both the Ks and Ka/Ks, and the GC2, GC3, and Fop also affected these three values in paralogs and orthologs to some extent.

### The expression patterns of ***V. fordii*** R2R3-MYBs in different tissues

**Fig. 5:**
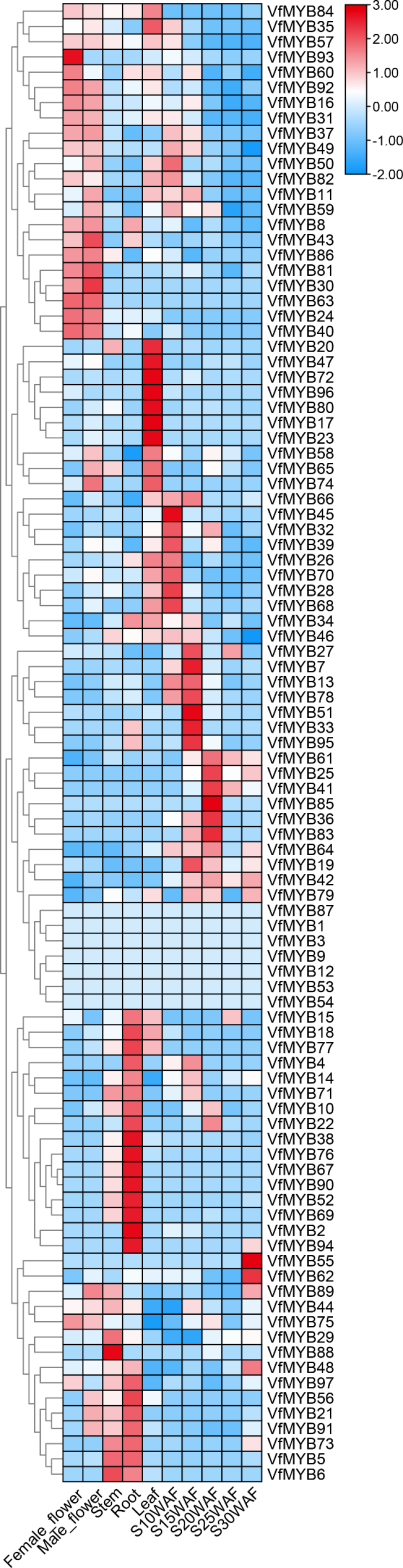
Expression levels of all *VfMYBs* in different tissues. Red and blue indicated high and low expression levels, respectively

**Fig. 6:**
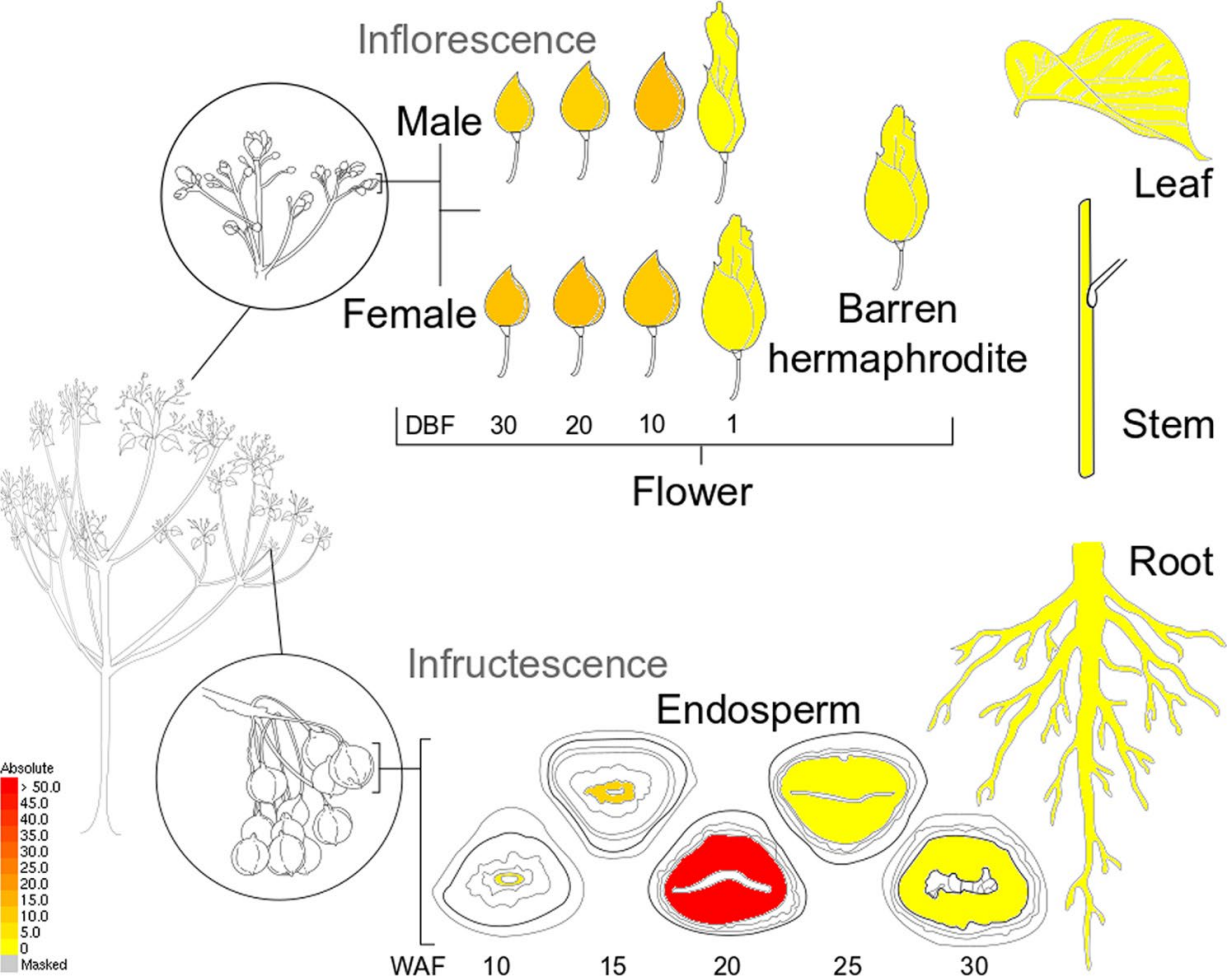
Electronic fluorescent pictographic (eFP) map of *VfMYB36* in different tissues or developmental stages. Red and yellow indicated high and low expression levels, respectively

The *MYB* family members are thought to play key roles in many aspects, such as stress responses, and plant growth and development [7, 8, 14, 50, 51]. To get more about the possible functions of *MYB* genes in different tissues or during fruit development, we used previously publicly available RNA-seq data from our lab to investigate the transcriptional abundance of *VfMYB* genes in *V. fordii* [48]. As shown in Fig. 5, the expression patterns of *VfMYBs* were tissue-specific in *V. fordii*. Among them, most *VfMYBs* showed tissue-specific expression patterns, such as *VfMYB20, VfMYB47, VfMYB73, VfMYB96, VfMYB80, VfMYB17*, and *VfMYB23* in leaves, *VfMYB2* and *VfMYB94* in roots, indicating that these meristem-specific *VfMYBs* might play key roles in cell fate specification and organ formation. These results highlighted the functions of *VfMYBs* in meristem tissues and provided a basis for further study of functions. In our laboratory, we investigated the patterns of oil synthesis in *V. fordii* that the oil content was relatively low during the initial stages of fruit development (10 weeks after flowering: WAF), but then rose rapidly until the middle stages (20–25 WAF) [48]. Finally, the oil content gradually decreased as the fruit matured [48]. This pattern was positively correlated with the expression pattern of the *VfMYB36* from subgroup C48 (Fig. 3) in developing seeds. The expression of this gene increased during the early to middle stages and then decreased as the seed matured (Fig. 6), suggesting *VfMYB36* might regulate seed-related traits. Indeed, this expressed gene *VfMYB36* (subgroup C48) was an orthologous gene of *A. thaliana AtMYB89*, which can regulate seed oil accumulation [52]. Therefore, we selected *VfMYB36* as the potential target for further study on the regulation of seed oil accumulation.

### ***VfMYB36*** involved in seed oil accumulation

**Fig. 7:**
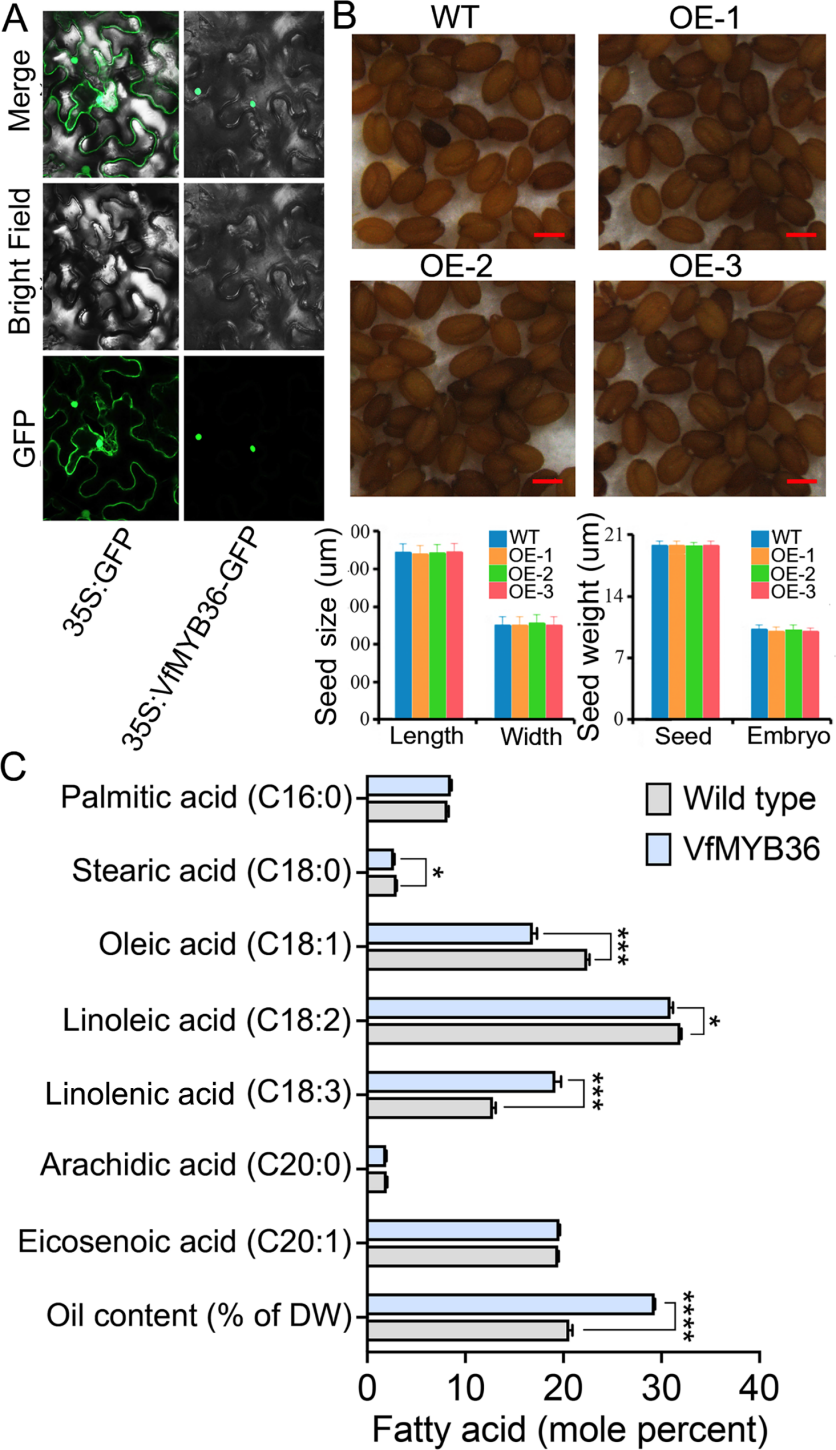
Functional analysis of *VfMYB36* in *V. fordii*. **A**, Subcellular localization of the VfMYB36 fused with GFP (35 S:VfMYB36-GFP) in tobacco leaves. **B**, Microscopic observation of mature seeds randomly selected from Wild type (Col-0) and *VfMYB36* overexpression plants. **C**, The content of major fatty acid (FA) species and oil in mature seeds of Wild type (Col-0) and *VfMYB36* overexpression plants

The nuclear localization of transcription factors plays a key role in their performance of regulatory functions [53]. The nuclear entry process that requires one or more nuclear localization signals is affected by many factors, such as developmental stage, cell cycle, and environmental pressure. MYBs are usually located in the nucleus. In our study, we constructed the pEaryleyGate104-VfMYB36 for examining the subcellular localization of VfMYB36 (Fig. 7A). We found that the green fluorescent signal from the expressed fusion of pEaryleyGate104-VfMYB36 was observed specifically in the nucleus. However, green fluorescent from the control vector was distributed throughout the whole cell (Fig. 7A).

In China, the seeds from *V. fordii* have been used to produce tung oil for more than a thousand years [54]. *MYB* genes have been confirmed to play important regulatory roles in the biosynthesis of seed oil [52, 55]. In our study, the *VfMYB36* was further selected to overexpress *A. thaliana* to study its role in oil biosynthesis. As shown in Fig. 7B, there were no significant differences in seed morphology and shape between overexpressed plants and wild-type plants, such as seed coat color, seed size, and dry weight (Fig. 7B). However, the overexpression of *VfMYB36* in *A. thaliana* resulted in a significant increase in the oil content (higher by 42.29%) of *A. thaliana* seeds (Fig. 7C). We further carried out the GC analysis to survey whether the overexpression of *VfMYB36* in transgenic *A. thaliana* causes any qualitative change in fatty acids types. Remarkably, we found that the linolenic acid C18:3 was increased by 49.61% in transgenic lines expressing *VfMYB36* compared with WT plants. C18:3 is a basic substance that constitutes cell membranes and biological enzymes, and is also an essential nutrient for people [56]. *AtMYB89*, an orthologous gene *VfMYB36*, overexpression decreases seed oil accumulation in *A. thaliana* [52], but interestingly *VfMYB36* overexpression in *A. thaliana* leads to increased linolenic acid (C18:3). To further understand the reasons for this difference, we selected six genes (*AtWRI, AtENO1, AtBCCP1, AtKAS1, AtKCS11*, and *AtPAL2*) related to oil biosynthesis that have been reported before [52], and detected their expression patterns between transgenic and WT plants. As shown in Figure S3, we found that the overexpression of *VfMYB36* can enhance the expression of these six genes, while the overexpression of *A. thaliana AtMYB89* mainly repressed the expression of these genes [52], possibly due to functional divergence of *MYB* homologs that evolved from a common ancestor. Therefore, our research not only provided a target for the production of biomass diesel, but also provided a convenient way for breeding germplasm resources with high linolenic acid content.

## Conclusion

Here, we identified the R2R3-MYBs in five Euphorbiaceae species and compared the gene numbers among these genomes. The phylogenetic analysis suggested that the majority of subfamilies had R2R3-MYBs from Euphorbiaceae species and *A. thaliana*, implying that the functions of most R2R3-MYBs are conserved and that genes within a given subfamily share recent common evolutionary origins during evolution. The paralogous and orthologous R2R3-MYB genes evolve at different rates. WGD or SD MYB genes exhibit gene expression divergence under normal growth conditions in *V. fordii.* Combining expression patterns with transgene analysis, our study provided a target for the production of biomass diesel and a convenient way for breeding germplasm resources with high linolenic acid content in the future.

## Methods

### Identification of R2R3-MYBs in Euphorbiaceae

Genome resources of *R. communis* were downloaded from the Phytozome (https://phytozome.jgi.doe.gov/pz/portal.html) database, the *J. curcas* was derived from Jatropha genome database (http://www.kazusa.or.jp/jatropha/), the *M. esculenta* was obtained from Ensembl database (http://plants.ensembl.org/Manihot_esculenta/Info/Index), the *H. brasiliensis* was downloaded from NCBI database (https://www.ncbi.nlm.nih.gov/). The *A. thaliana* R2R3-MYBs were obtained from a previous study and the corresponding sequences were derived from TAIR database (www.arabidopsis.org/) [18]. Proteins with a MYB domain (PF000249) were identified by the hidden Markov model using HMMER v3.2.1 software and BlastP (E-value = 1e -3) similarity searches in five Euphorbiaceae genomes: *R. communis*, *J. curcas*, *M. esculenta*, *H. brasiliensis* and *V. fordii* [57]. Subsequently, all obtained MYB sequences were again detected using Pfam database (http://pfam.xfam.org/) [58], InterPro (http://www.ebi.ac.uk/interpro/scan.html) [59], and SMART (http://smart.embl-heidelberg.de/) [60] for domain structure of MYB (PF000249). Finally, we discarded those MYB sequences that lack core or complete MYB domain (PF000249).

### Phylogenetic analysis of R2R3-MYBs

The multiple sequence alignment of all full-length R2R3-MYBs was performed using MAFFT 7.0 software [61]. ModelFinder was used to estimate the best substitution model for R2R3-MYBs with maximum likelihood (ML) [62]. IQ-TREE software was used to construct the ML tree using ultrafast bootstrap approximation with 1000 and SH-aLRT test set to 1000 random addition replicates [63]. Finally, the tree was visualized with online iTol (https://itol.embl.de/) [64] and FigTree software.

### Synteny and positive selection analysis of R2R3-MYBs

Paralogs are genes that are related by duplication within the same genome [65, 66]. Orthologous genes are often defined as genes that have a synteny relationship in different genomes, according to previous studies [65–67]. The intra-species and interspecies synteny analyses were performed using a similar step to that developed for the PGDD [46]. First, we searched the potential homologs using BLASTP software (E < 1e-10, top five matches). Then, these results as the input object for MCScanX software were used to confirm syntenic chains with the following parameters: Max GAPs, 25; E-value, 1e-05; Overlap window, 5; GAP penalty, − 1; Match score, 50; Match size, 5 [68]. Further, the “duplicate_gene_classifier” script in the MCScanX software was also used to identify the various types of duplications, including WGD/segmental, tandem, proximal, and dispersed duplication events. The KaKs_Calculator 2.0 software was used to estimate the synonymous substitution rates (Ks) and nonsynonymous substitution rates (Ka) [33]. If the Ka value was nearly 0, and the Ks value was more than 3 or less than 0.01, these duplicated genes were excluded, because a high Ks value implied the risk of saturation, and low sequence divergence could lead to unknown results. When Ka/Ks < 1, >1, and = 1, it suggested purifying selection, positive selection, and neutral selection, respectively.

### Gene feature in homologs

The gene features, including the frequency of optimal codons (Fop), polypeptide length, and GC content at three codon positions (GC1, GC2, and GC3), were estimated to compare duplicated and singleton R2R3-MYB genes. The CodonW 1.4 (http://codonw.sourceforge.net) was used to estimate the polypeptide length and Fop. The in-house perl script was carried out to calculate the GC content.

### ***Vernicia fordii*** RNA-seq data analysis

The RNA-seq data (BioProject: PRJNA503685, PRJNA445350, and PRJNA483508) were collected and retrieved from SRA database, and then these raw data were processed by trimmomatic with default parameters [69]. The HiSAT2 [70] was carried out to align the RNA-Seq clean reads to the masked *V. fordii* genome, and then the Stringtie [70, 71] was used to obtain the Fragments Per Kilobase Million (FPKM) values, as described by Cao et al. (2021) [72].

### Subcellular localization

The primer VfMYB36-F, 5’-ATGGAGAGTAAGCAGTCAAAAG-3’ and VfMYB36-R 5’ TCAAGAGGCTCCTACTCCAAG-3’ was used to clone the *VfMYB36* from *V. fordii* seeds. Then we used the Gateway system to produce the pEaryleyGate104-VfMYB36 for subcellular localization. The Gene Pulser Xcell (BIO-RAD, country-region USA) was used to electroporate pMDC32-VfMYB36 into Agrobacterium tumefaciens GV3101. Use the injection method to infiltrate the suspension into the tobacco leaves. The confocal laser scanning microscopy (CarlZeiss LSM710, Germany) was implemented to observe the expressed YFP signal.

### Fatty acid analysis of ***A. thaliana*** over-expressing ***VfMYB36***

The Gateway system was used to produce the over-expression vector pMDC32-VfMYB36 for the transgenic experiment. We transformed the pMDC32-VfMYB36 vector into *A. thaliana* by Agrobacterium-mediated the floral dip method [73]. Based on the protocols described by Chen et al. (2010), the gas chromatograph GC-2014 (Shimadzu, Japan) was used to measure all fatty acids [74]. Three biological repeats of all experiments were performed in our study. GraphPad Prism v9 was used for data analysis and visualization.

### qRT-PCR analysis

The MiniBEST Plant RNA Extraction Kit (TaKaRa) was used to extract total RNA, and the PrimerScript RT (TaKaRa) was carried out for reverse transcription. The SYBR Green Master Mix (TaKaRa) was used to perform the qRT-PCR for three biological replicates. The primers for qRT-PCR using Primer-BLAST (https://www.ncbi.nlm.nih.gov/tools/primer-blast/index.cgi; Table S6). A 2^–ΔΔCt^ method was used to calculate the relative expression level as described previously [13, 75].

## Electronic supplementary material

Below is the link to the electronic supplementary material.

Supplementary Material 1

Supplementary Material 2

Supplementary Material 3

Supplementary Material 4

Supplementary Material 5

Supplementary Material 6

Supplementary Material 7

## Data Availability

Expression data of *Vernicia fordii* were available in NCBI SRA database with accession numbers: PRJNA318350, PRJNA483508, and PRJNA445068.

## Abbreviations

Ks: synonymous substitutions per site
HTH: helix–turn–helix
CDS: coding sequence
DBF: day before flowering
GFP: green fluorescent protein
DIC: bright field image
WAF: week after flowering
AMV: avian myeloblastosis virus
Ka: nonsynonymous substitution rates
