# Supplementary material for: Large-scale analysis of putative Euphorbiaceae R2R3-MYB transcription factors identifies a MYB involved in seed oil biosynthesis
Source: BMC Plant Biol. 2023 Mar 17;23:145. doi: 10.1186/s12870-023-04163-5 (PMC10022305; doi:10.1186/s12870-023-04163-5)
Supplement: Supplementary file 1 — Supplementary Material 1 [file 12870_2023_4163_MOESM1_ESM.docx]

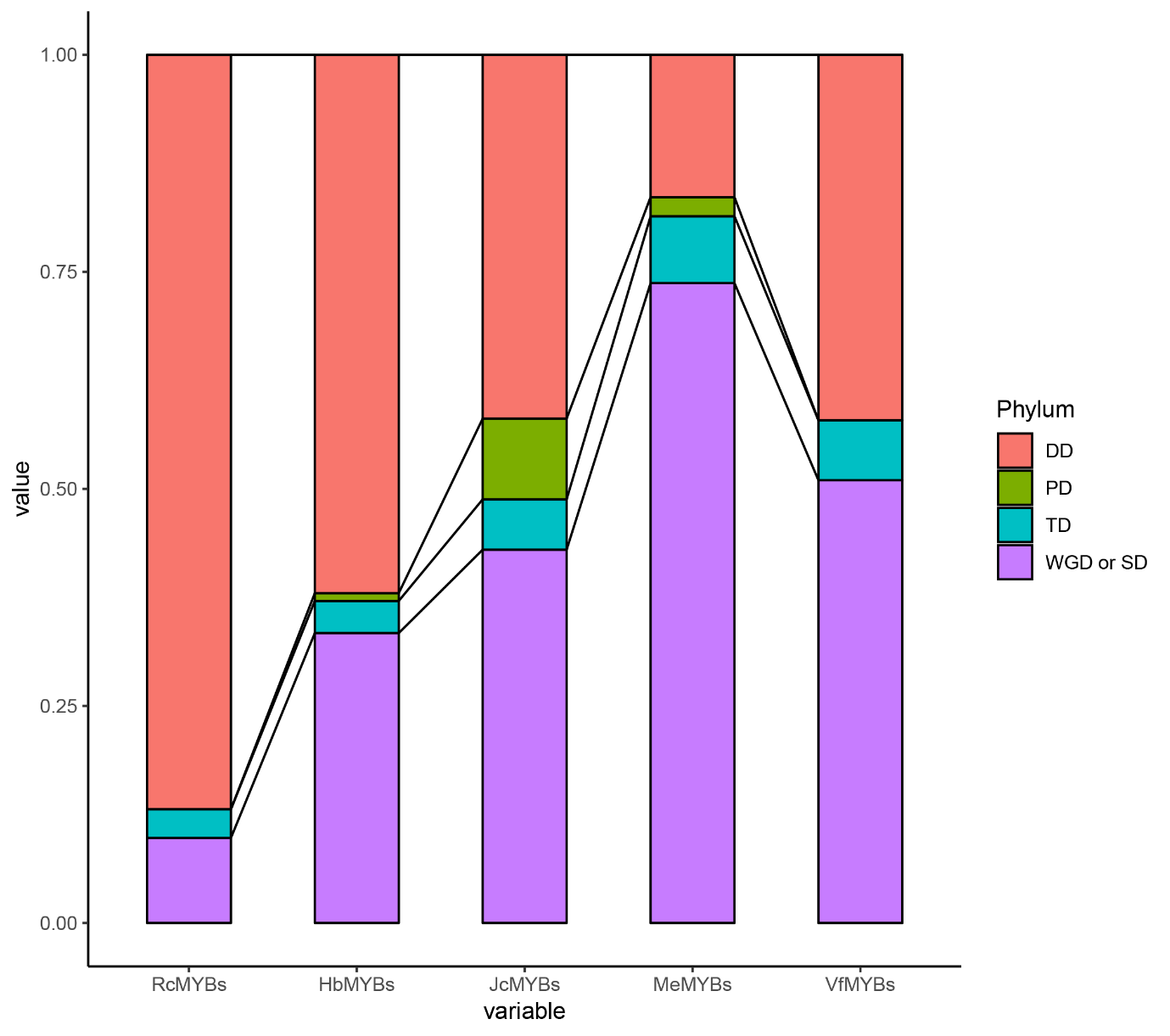


Figure S1. The number of DD, PD, WGD/SD, TD of MYB genes in Euphorbiaceae genomes. Different bars indicated the duplicated genes, and each color represented each Euphorbiaceae genome.


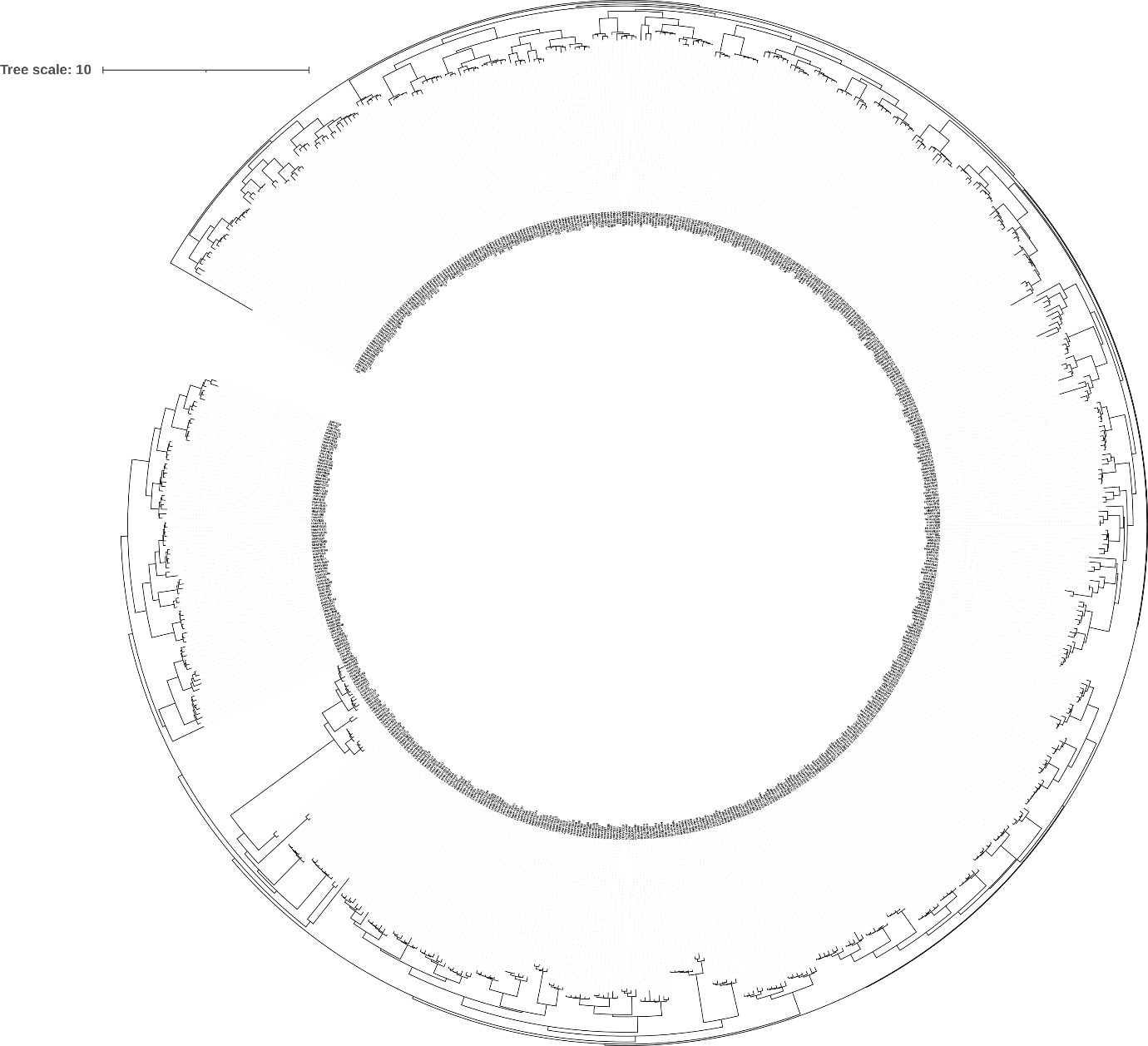


Figure S2. The ML tree of MYB genes from these five Euphorbiaceae species. This tree was constructed using IQ-tree with the best substitution model.


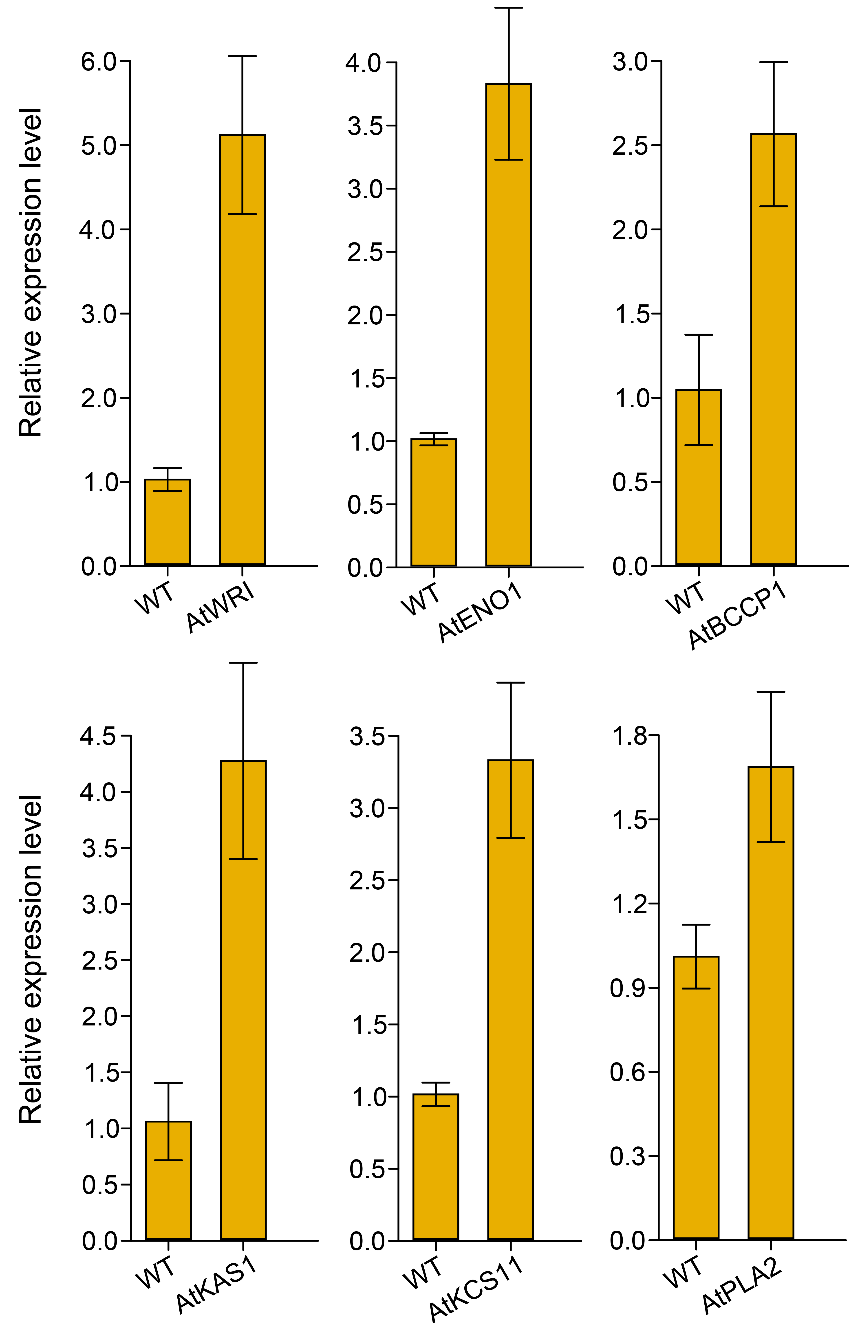


Figure S3. Expression levels of oil synthesis related genes genes in WT and transgenic plants
